# Dr. Isaac E. Taylor

**Published:** 1889-12

**Authors:** 


Dr. Isaac E. Taylor, of New York, died suddenly of pericarditis, at his home, October 30, 1889. Dr. Taylor has been prominent in medical circles for more than thirty years, and his death is lamented by an extensive professional and lay acquaintance, besides immediate relations and personal friends.

